# Online Mindfulness Experience for Emotional Support to Healthcare staff in times of Covid-19

**DOI:** 10.1007/s10916-022-01799-y

**Published:** 2022-01-26

**Authors:** Gema Castillo-Sánchez, Olga Sacristán-Martín, María A. Hernández, Irene Muñoz, Isabel de la Torre, Manuel Franco-Martín

**Affiliations:** 1 Department of Signal Theory and Communications, and Telematics Engineering, Universidad de Valladolid, Valladolid, Spain; 2 RedIAPP (Primary Care Prevention and Health Promotion Research Network), Valladolid, Spain; 3 Río Hortega University Hospital, Valladolid, Spain; 4 Zamora Healthcare Complex, Zamora, Spain

**Keywords:** Mindfulness, Online, COVID-19, CSQ-8, Survey, Mental health, Spain

## Abstract

During the first confinement in Spain, between the months of March to June 2020, Information and Communication Technologies strategies were implemented in order to support health workers in the Wellbeing of Mental Health. Faced with so much uncertainty about the pandemic, an Online Mindfulness course. The objective of the course was to support healthcare professionals in Castilla y León in managing stress, anxiety and other emotional disturbances generated by coping with a situation as uncertain and unexpected as a pandemic, in order to manage emotions and thoughts that can lead to suicidal ideation. The motivations for the demand, reasons or motivations in which the health professionals of Castilla y León decided to participate in the mindfulness course in the first wave of Covid-19 in Spain are described. The descriptive and inferential statistical analysis of the customer satisfaction survey applied at the end of the mindfulness course, to the health professionals who participated in a satisfaction survey (CSQ-8: Client Satisfaction Questionnaire). Professional were asked to complete a survey based on (CSQ-8: Client Satisfaction Questionnaire) whose Cronbach's alpha = 0.917 is why the instrument used with N = 130 participants has high reliability. The 66% answered with a highly satisfied that they would return to the mindfulness online course. The 93% of the people who answered the satisfaction survey were women, of which they are professionals in the nursing area, with a participation of around 62%. In relation to the online system used in the Mindfulness intervention, 74% expressed that they fully agreed that it has been easy to use the online system for the mindfulness intervention. Health Professionals responded with 58% high satisfaction and 36% satisfaction, making a total of 94% on the help received in the online mindfulness courses to solve their problems. There is no difference between the age groups of the professionals who have preferred the Mindfulness online course (p = 0.672).

## Introduction

During the epidemic 2020, health workers on the front lines often come to a very high pressure for care, including increased risk of infection and contamination, as well as frustration, work overload, isolation, management of patients and families with negative feelings, due to risk of contagion [1, 2].

In addition, the fear of infecting their relatives through their work, which in many cases leads them to isolate themselves from the family or loved ones [2]. Even, in previous experiences, it has been seen that the risk of post-traumatic stress disorder increases, when the professional's quarantine is associated with the infection of a family member [3].

In the treatment of the epidemic caused by covid-19 [4] The Chinese experience has frequently shown that stress and panic reactions occur in both health workers and affected patients [2, 5].

The importance that goes beyond mental suffering and loss of performance, but will also generate a decrease in the immune protection capacity of their own body that will expose them to a greater risk of infection [6]. Some studies show a high suicide rate in the group of health professionals [7], which is being particularly affected and at risk in the battle against COVID-19. This situation fosters a global debate about the concerns of healthcare professionals about the spread and management of infection, exposure of family members, sick colleagues, and work stress [8]. This group of professionals deserves emotional support services in their work, which is why the initiative arises to offer Mindfulness [9] online.

During the Covid-19 pandemic there are innovative opportunities for the use of technologies to maintain social distancing, monitor mental health [10] and assist the sick [11]. Mobile health services appear to be highly reliable for digital psychological intervention [12].

A high prevalence of psychological distress has been estimated in professionals facing the pandemic [13] and psychological disorders associated with the situation [14], as well as stress affects their behavior [15]. This study presented an immense literature pertinent to the prevalence and diagnosis of stress in humans [16]. It is advisable to support the work of professionals and mental health should be monitored regularly using emerging technologies in times of pandemic [17].

Some interventions, such as Mindfulness-Based Cognitive Therapy (MBCT) [18] have shown that they can protect people from depression [19] and can also facilitate a change in self-regulation goals to prevent suicide [20].

During the first wave of the pandemic, health professionals in Castilla y León (Valladolid and Zamora) used this online therapy as support for the management of stress [21], anxiety [19] and other alterations in emotional and mental health contributing directly to suicide prevention [7, 8, 22].

In this article we aim to assess the degree of satisfaction and results of a first approach to the intervention through mindfulness applied online to promote emotional control of the professionals of the Health staff of Castilla y León. The remainder of this paper is a follows methods, results, discussion and conclusions and how this may apply to other health care employees.

## Methods

In the first confinement in Spain during the months of March to June 2020, strategies were organized involving Information and Communication Technologies [23] to be able to support health workers in the Mental Health Wellness. Faced with so much uncertainty about the pandemic, an Online Mindfulness course [9] was organized using the ZOOM videoconference platform [24] and published on the website www.massaludmental.es [25] for healthcare professionals in Castilla y León. The objective of the course was to support healthcare professionals in Castilla y León in managing stress, anxiety and other emotional disturbances generated by coping with a situation as uncertain and unexpected as an outbreak, in order to manage emotions and thoughts that can lead to suicidal ideation.

This initiative began on April 20 and ended on June 28, 2020. In total, 24 sessions of 40 min duration were held, twice a week, in the morning and afternoon shift online.

The organization of this initiative included as a first stage the online registration to be able to send them by mail the links of the course and the planning of sessions. Through enrollment, questions were asked related to the motive and reasons that prompted them to participate in the mindfulness course. All was offered free for health workers.

The methodology used to assess the impact of mindfulness on health professionals in Castilla y León, consists of the following steps:

- The motivations for the demand, reasons or motivations in which the health professionals of Castilla y León decided to participate in the mindfulness course in the first wave of Covid-19 in Spain are described. The number of registered is equal to N = 359 professionals. However, with regard to the question about the reason for the lawsuit, 340 responded. Details on the registration of participants can be found in the appendix in Table 7.
- The descriptive and inferential statistical analysis of the customer satisfaction survey applied at the end of the mindfulness course, to the health professionals who participated in a satisfaction survey (CSQ-8: Client Satisfaction Questionnaire) [26], in which they could answer in an anonymous and not mandatory. The number of professionals who answered is equal to N = 130. Details on the satisfaction survey applied can be found in the appendix in Table 8.

The descriptive and inferential statistics was carried out with samples collected for non-probabilistic convenience [27]. This was due to the ease of access and the availability of the professionals who participated in the mindfulness course. The tool used for the analysis was the Statistical Package for Social Sciences 24 (SPSS). The analysis of the open questions of this course have been published in another article [28].

## Results

A total of 359 health professionals participated, mostly belonging to the Zamora and Rio Hortega University hospitals, there were participants from all over Spain, mainly from Castilla y León, reaching a total of 359 participants. The main statistical data obtained on the registration of the course are presented in Tables 2, 3, and 4:

According to Table 1, the professionals at the beginning of the first wave of the pandemic demanded this type of intervention, the main reasons, for which the health professionals required this course were: Anxiety (124), Insomnia (38), Depressive mood (12), psycho-emotional support (140) and others (26). Additionally, work stress was reported by 213 registered participants, followed by stress for relatives (patients with Covid-19) with 10, stress due to covid contagion reached 41. As for the other professionals, in this group we find economists, engineers, unemployed, self-employed, civil servants, laboratory technicians, with N = 340.

In Table 2, 93% of the people who evaluated the course were women. Table 3 shows us the variety of professionals interested in the Mindfulness course, 53% were from the nursing / TCAE area, the percentage from Hospital-others refers to 20%. The hospital workers that did not identify role group totaled 14%. Finally, health professionals such as Doctors represent 13%.

**Graph 1:**
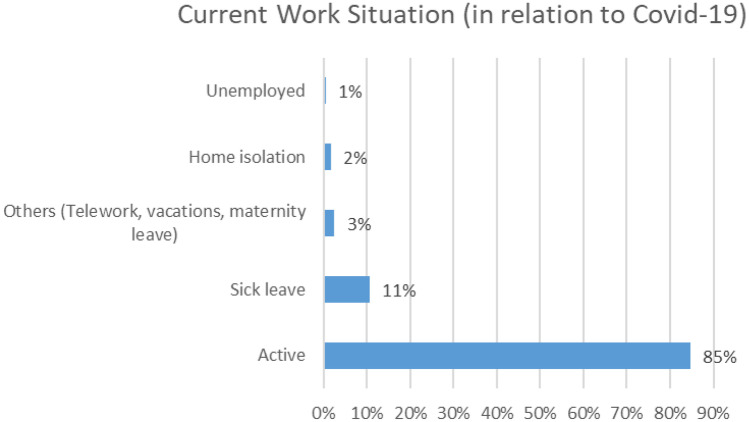
Current Work Situation

According to Graph 1, 85% of the professionals who enrolled in the course were active in the care of their functions, 11% were on sick leave, 3% reported that they were Others (teleworking, vacations, maternity leave, 2%, home isolation and another 1%, unemployment situation. Registered professionals are in a range of 27 to 63 years.

In state final of Mindfulness course, professionals were asked to complete a survey based on (CSQ-8: Client Satisfaction Questionnaire) [26] voluntarily and anonymously, whose Cronbach's alpha = 0.917 is why the instrument used with N = 130 participants has high reliability and the general characteristics of the professionals are presented in Table 4.

In Table 4, the 93% of the women answered this survey, of which 62% are professionals in the nursing area and the rest of the professionals who took advantage of the course were doctors 12%, others related to health with 26%.

According to Table 4, some workplace of healthcare professionals in the Critical Care Unit (UVI / REA) with 8%, Primary Care with 10%, 35% belonging to other professionals within the Hospital and 21% to administration professionals, related technicians with the health sector.

We also observe that by age ranges, 42% of the participating professionals are between 36 and 45 years old and 29% are between 46- and 55-years old professionals with experience by Table 4.

Overall, the health professionals who responded to the mindfulness course satisfaction survey indicated the following in Table 5 (CSQ-8 items detail in the appendix Table 8).

According to Table 5, shows question C1, 62% of the health professionals evaluated the course as highly satisfied and Satisfied with 35%. In relation to C2, Professionals indicated 47% highly satisfied and Satisfied with 49% on the class of service required, they fulfilled what was required.

In relation to C2, Professionals indicated 47% highly satisfied and Satisfied with 49% on the class of service required, they fulfilled what was required. In C3, where they asked whether the course had helped them solve their problems (emotional distress), 21% highly satisfied and Satisfied with 46% who indicated that for the most part this course has helped them solve their problems. In this question, 33% indicated dissatisfied.

In C4, 65% indicated that they would recommend the program if a friend was in the same need for help and 33% indicated that they would recommend it for a level of satisfied.

With regard C6, the services received helped him face his problems was evaluated with 58% high satisfaction and 36% satisfaction, making a total of 94% of professionals who helped them mindfulness by Table 5.

In C7, the professionals indicated a high percentage of satisfaction with the course, with 70% of the service received, which validates what was answered in C2. As answered in C8, 66% answered with a highly satisfied that they would return to the mindfulness program, which corresponds to what was answered in C4 and C1 by Table 5.

According to Table 6, we can observe important aspects about the mindfulness course applied to health professionals during the first Wave of Covid-19. When asked if the duration of the intervention was short, the professionals responded with 10% in complete agreement, 39% in agreement and 21% were not sure, 25% in disagreement, which indicates that only 5% considered that the duration was short, the rest were not.

The degree of privacy and respect for privacy was considered with 65% and 30% completely and in agreement respectively, as an action that was considered and complied with. The sessions were carried out with Zoom at the scheduled times, and with access by password.

In relation to the online system used in the Mindfulness intervention, 74% expressed that they fully agreed that it was easy to use the online system for the mindfulness intervention by Table 6.

**Graph 2:**
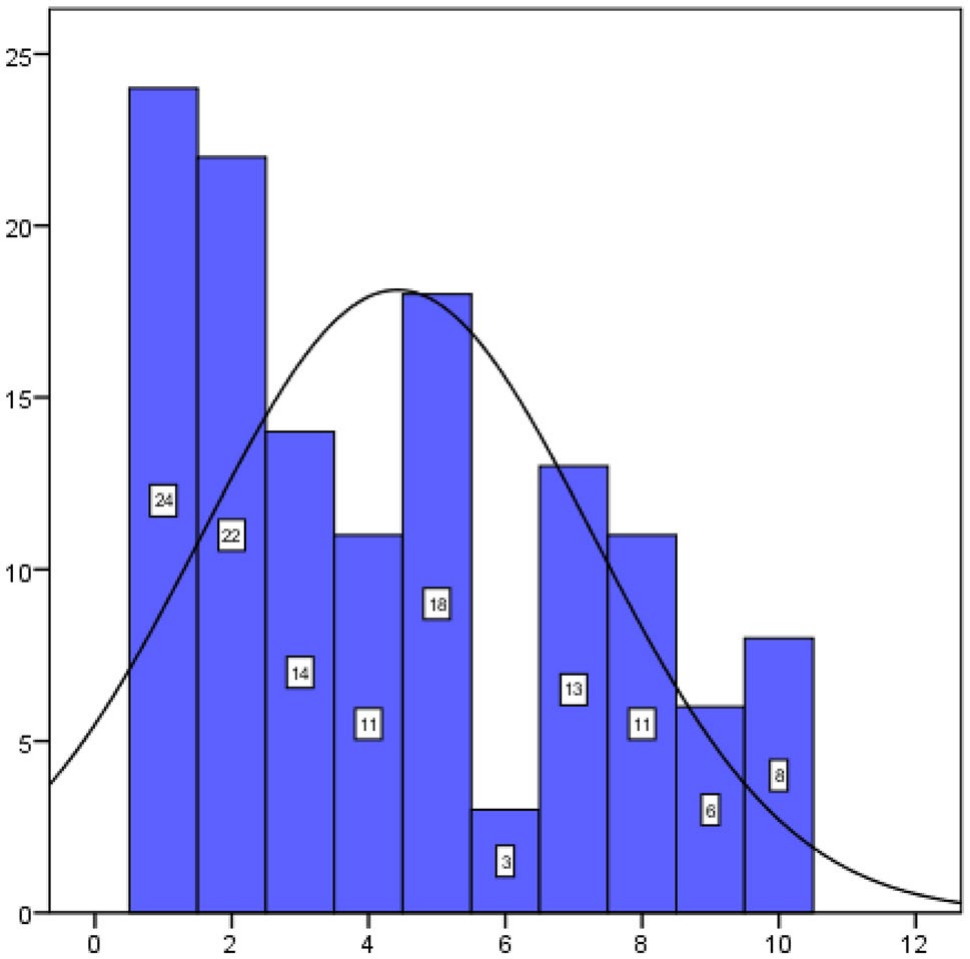
Mindfulness course intervention preferences (1 = Online, 5 indifferent, 10 Face-to-face)

According to Graph 2, where we compare the face-to-face (face-to-face) and online intervention by means of a linear scale, we can see that the majority select the online option when approaching the values 1 and 2, over the face-to-face one.

**Graph 3:**
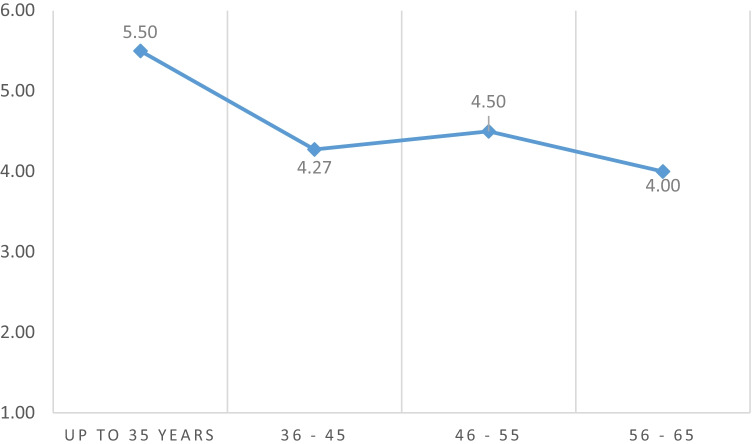
Difference of means by age groups

By making associations between the Mindfulness course preference intervention with the following variables, we obtain the following:

- When verifying if there is a difference between the age group of the participating professionals, the H. Kruskal -Wallis statistical test is carried out, obtaining p = 0.672 > 0.05, we can conclude that there is no difference between the age group with 95% confidence. We can also observe Graph 3.
- To check if there is a difference between the group of type of professionals, the H. Kruskal -Wallis statistical test is carried out, obtaining p = 0.001 < 0.05, we can conclude that there is a difference between types of professionals.
- We associate with workplace of participating professionals, the H. Kruskal -Wallis statistical test is carried out, obtaining p = 0.01 < 0.05, we can conclude that there is a difference between workplace of professionals with 95% confidence.

## Discussion

The uncertainty and work overload during the pandemic in the first wave of Covid-19, led health professionals in many cases to be affected by emotional distress.

Given the high demand for Support, the need to maintain privacy and the stress that the hospital generated in itself, as well as the extension of hours in the hospital, led the Research team to carry out an intervention program based on mindfulness intervention that could meet the requirements and demands collected:

- Easy accessibility.
- Preserve privacy.
- Intervene for emotional improvement without prejudging any previous mental pathology or during the intervention.

Due to the experience in the mindfulness intervention of one of the authors (OS-M), this type of intervention was proposed, which had shown its efficacy in various settings [29–31], although it was difficult to apply it in people, who were sometimes doing 12-h shifts, motivated us to offer the course in an online application format, through the use of new technologies and supported by some studies that showed great possibilities for this type of application [32, 33].

The first issue to highlight is the high number of participants, 359, which is an unthinkable number to reach so quickly with a traditional application, which would also be significantly more expensive. Considering that the diffusion had been limited and that it was being a pilot project, this high number was not surprising. In fact, we have not found studies of this type with such a large number of participants.

Also although the study design of being online was optimal during a pandemic wave, this may or may not be comparable to traditional models of supportive care [34].

The greatest demand for the course is among women and nursing workers, which could lead to the assumption that this type of intervention should be fitted mainly to women and nurses for being the main attenders, which should also be considered as a measure tending to provide a preferential service to women, about 93% are preferred by women. According to Graph 1, 85% of the professionals who enrolled in the course were active in the care of their functions and 11% were on sick leave, what means that this kind of approach can be useful for preventing to get sick but can be a good support for people already sick demanding a psychological support. Considering the figures and acceptation/satisfaction of the therapy it should be considered as probably the best tool for facing to the emotional problems in the health works linked to the outbreak.

The challenge then was based on achieving satisfaction for the attendees both in relation to the program and its means of application. In general, psychologist and therapist are reluctant to the online interventions [35] and so, it was important to know the level of acceptation and usability of this online approach [36]. Then it is observed that in general the degree of acceptance by the attenders is very high, with a very good interactive response and that there is even a general preference for its application online over the face-to-face route. This is influenced by privacy, comfort and avoidance of spending more time in the hospital where they may have had 12-h shifts. So, despite of the barriers of implementing this kind of therapies, it´s good to try them more because users welcome them with high satisfaction [37].

In general, 62% of health professionals evaluated the course as excellent and Good with 35%, which in total allows us to say that 98% indicate that there is great satisfaction about the application of this type of therapy by C1, with many cases in which the acceptance of it improved compared to traditional treatments, which leads to consider that online intervention should be considered in cases, such as the present, in which the demand is great, the resources are scarce and social distance is maintained. Indeed, traditional videoconferencing system has proved sufficient capacity to be able to connect with health professionals, as well as the possibility of greater participation since it can be followed from any point, rising the accessibility.

## Conclusions

Participants were asked to complete a survey based on (CSQ-8: Client Satisfaction Questionnaire) [25] voluntarily and anonymously, whose Cronbach's alpha = 0.917 is why the instrument used with N = 130 participants has high reliability.

The 93% of the people who answered the satisfaction survey were women, of which they are professionals in the nursing area, with a participation of around 62%.

The 62% of the health professionals evaluated the course like highly satisfied and Satisfied with 35% by question C1. The 65% indicated that they would recommend the program if a friend was in the same need for help and 33% indicated that they would recommend it for a level of satisfied.

Health Professionals responded with 58% high satisfaction and 36% satisfaction, making a total of 94% on the help received in the online mindfulness courses to solve their problems.

The degree of privacy and respect for privacy was considered with 65% and 30% completely and in agreement respectively, as an action that was considered and complied with.

In relation to the online system used in the Mindfulness intervention, 74% expressed that they fully agreed that it has been easy to use the online system for the mindfulness intervention.

According to the Kruskal–Wallis test, there is no difference between the age groups of the professionals who have preferred the Mindfulness online course (p = 0.672).

Finally, this online mindfulness experience was organized spontaneously by professionals who had the initiative to innovate in times of COVID-19 and to provide mental support to professionals to prevent risks associated with their workload. Although this approach was used due to the pandemic as a way to offer supportive services, the implications for practice as we move from the pandemic may meet healthcare workers needs of support in a convenient way through online programs.

Future work will continue to provide more mindfulness courses to support health personnel in the COVID-19 pandemic.

**Table 1:**
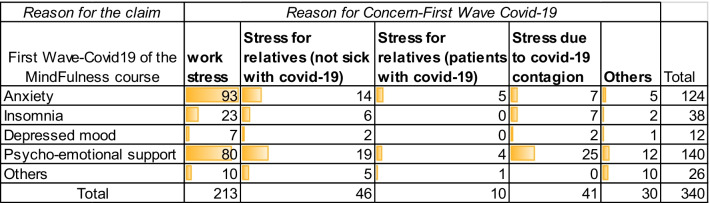
Distribution of the frequencies of the variables considered in the Reason and demand of the Course

**Table 2:**
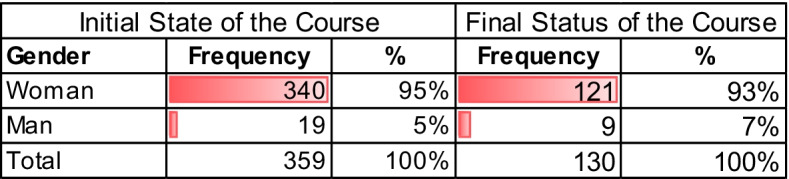
Frequency distribution—by Sex (initial state and final time) of the Mindfulness course participants

**Table 3:**
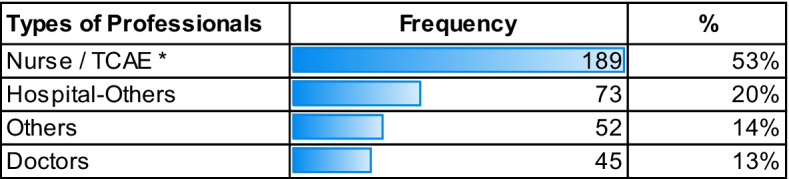
Distribution of the frequencies of the types of health professionals

*Nursing Auxiliary Care Technician (TCAE)

**Table 4:**
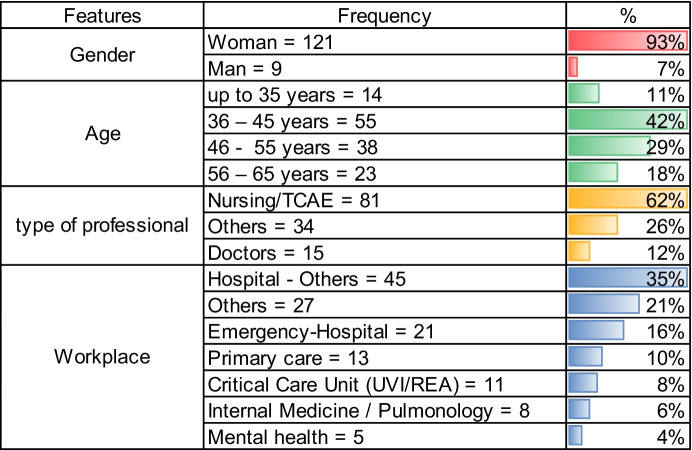
General characteristics of the professionals

**Table 5:**
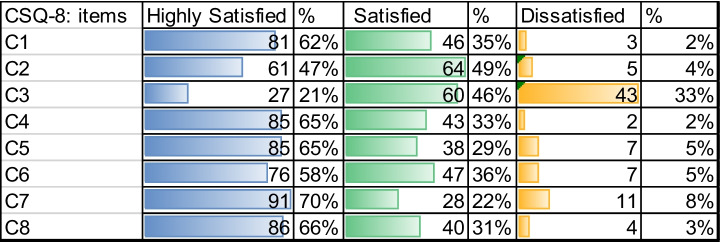
Frequency distribution of client satisfaction questionnaire by each question in Castilla y Leon, 2020 (n = 130)

**Table 6:**
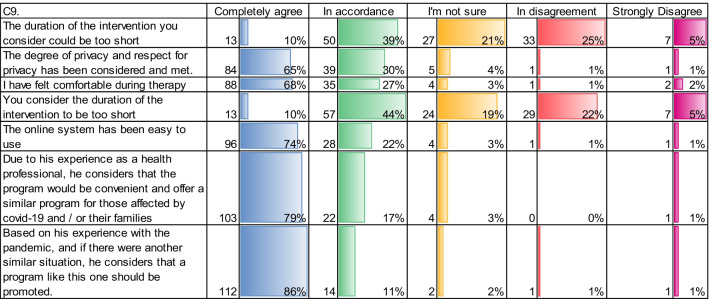
C9. Evaluation of General aspects in terms of Quantity and Percentages of the Mindfulness Course

## Appendix

**Table 7:** Registration to the Mindfulness Course in time of the COVID-9 Pandemic

ID	Question	Answers
Item 0	E-mail	Email
	Name and surname	Text
	Age	Numerical quantity
	Sex	woman man
	Workplace	Nurse / TCAE Doctor Hospital-other Hospital-emergencies
1	Knowledge of the program and type of referral:	By e-mail By Direct Contact By phone By the traditional system (Interconsultation) Social media
2	Current work situation (in relation to COVID-19):	Active Sick leave Home isolation Unemployed Others: telecommuting, maternity leave, freelance, vacations
3	Reason for the demand	Anxiety Insomnia Depressed mood Psycho-emotional support Others
4	Main reason for concern associated with Covid-19	Work stress Stress for relatives (not sick with covid-19) Stress for relatives (patients with covid-19) Stress due to covid-19 contagion others (Concern about the social consequences (unemployment, economy) of the pandemic, emotional control, uncertainty, proximity of vulnerable people, relatives, deceased by covid) Hostility between colleagues in the service …

**Table 8:** Survey based on Client Satisfaction Questionnaire (CSQ-8)

ID	Question	Answers
Item 0	Type of professional	text
	Age	Numerical quantity
	Gender	Woman Man
	Workplace	text
Item 1	1. How would you evaluate the quality of the mindfulness online emotional support service you have received?	Excellent Good Regular Bad
Item 2	2. Did you receive the kind of service you required?	No, definitely In very few Yes, in general Yes, definitely
Item 3	3. What extent has this program helped you to solve your problems?	In almost all For the most part Only in some None
Item 4	4. If a friend were in need of similar help, I would recommend this program	No, definitely No, I think No Yes, I think so Yes, definitely
Item 5	5. How satisfied are you with the amount of help you have received?	Not satisfied at all Indifferent or moderately dissatisfied Moderately satisfied Very satisfied
Item 6	6. Have the services you received helped you cope better with your problems?	Yes, they helped me a lot Yes, they helped me something No, they really didn't help me No, they seemed to make things worse
Item 7	7. Overall, how satisfied are you with the services you have received?	Very satisfied Moderately satisfied Somewhat Satisfied Very dissatisfied
Item 8	8. If you needed help again, would you return to our program?	No, definitely No possibly Yes, I think so Yes, for sure
Item 9	9. About the Course:	
	• [Do you think the duration of the intervention could have been too short] • [The degree of privacy and respect for privacy has been considered and complied with] • [I have felt comfortable during therapy] • [Do you think the duration of the intervention could have been too short] • [The online system has been easy to use] • [Due to his experience as a healthcare professional, he considers that the program would be convenient and offer a similar program for those affected by covid-19 and / or their families] • [Due to his experience with the pandemic, and if there were another similar situation, he considers that a program like this should be promoted.]	Completely agree In agreement I'm not sure In disagreement Strongly disagree
Item 10	10. Comparing the in-person (face-to-face) and online intervention, if you had to choose and after the lived experience, where would you place your preferences (A five [5] would be that you are indifferent for both types of interventions):	Scale [1 Online—10 face-to-face]
Item 11	Optional: -Share with us what you liked the most about the attention received -Share with us your suggestions and recommendations to improve	Open Answers
